# Identification of differentially expressed microRNAs as potential biomarkers for carcinoma ex pleomorphic adenoma

**DOI:** 10.1038/s41598-022-17740-9

**Published:** 2022-08-04

**Authors:** Hyojin Kim, Shin Eun, Woo-Jin Jeong, Soon-Hyun Ahn, Yun Jung Bae, Joong Seob Lee, Heejin Kim

**Affiliations:** 1 Department of Pathology, Seoul National University Bundang Hospital, Seoul National University College of Medicine, Seongnam, Republic of Korea; 2 Department of Pathology, Dongtan Sacred Heart Hospital, Hallym University College of Medicine, Hwaseong, Republic of Korea; 3 Department of Otorhinolaryngology-Head and Neck Surgery, Seoul National University Bundang Hospital, Seoul National University College of Medicine, Seongnam, Republic of Korea; 4 Department of Otorhinolaryngology-Head and Neck Surgery, Seoul National University Hospital, Seoul National University College of Medicine, Seoul, Republic of Korea; 5 Department of Radiology, Seoul National University Bundang Hospital, Seoul National University College of Medicine, Seongnam, Republic of Korea; 6 Department of Otorhinolaryngology-Head and Neck Surgery, Hallym University Sacred Heart Hospital, Hallym University College of Medicine, Anyang, Republic of Korea; 7 Department of Otorhinolaryngology-Head and Neck Surgery, Dongtan Sacred Heart Hospital, Hallym University College of Medicine, Hwaseong, Republic of Korea

**Keywords:** Cancer, Molecular biology, Biomarkers, Oncology, Pathogenesis

## Abstract

Carcinoma ex pleomorphic adenoma (CXPA) is a rare malignancy that transforms from PA. Early detection of the carcinoma by biopsy is difficult due to similar histopathology of the malignant and benign components. To address this, we investigated and compared the characteristic miRNA expression patterns across samples of the PA, carcinomatous portions (CA) of CXPA, as well as conventional PA. We selected 13 CXPA and 16 conventional PA FFPE samples, separated the PA and CA portions of CXPA samples and conducted miRNA profiling for each group. Among 13 transcripts that were differentially expressed between PA and CA of CXPA, eight miRNAs were up-regulated and five down-regulated in CA. Bioinformatic analysis revealed that the up-regulated miRNAs were related to cancer progression and down-regulated ones to tumor suppression. Additionally, seven miRNAs were significantly up-regulated in PA of CXPA compared to conventional PA, although they are histopathologically similar. Almost all of these transcripts interacted with TP53, a well-known tumor suppressor. In conclusion, we identified differentially expressed miRNAs in PA and CA of CXPA, which were closely associated with TP53 and various cancer-related pathways. We also identified differentially expressed miRNAs in the PA of CXPA and conventional PA which may serve as potential biomarkers.

## Introduction

Carcinoma ex pleomorphic adenoma (CXPA) is a rare malignant neoplasm of the major salivary glands, accounting for about 3–15% of all salivary gland malignancies^1,2^. In addition, CXPA has been shown to exhibit a rising incidence rate making it a growing concern for public health policy^3^. CXPA is the result of malignant transformation of the most common benign salivary gland tumor—pleomorphic adenoma (PA)—into various malignant histologies including salivary duct carcinoma (SDC), adenoid cystic carcinoma (AdCC), mucoepidermoid carcinoma (MEC), and myoepithelial carcinoma, amongst others^4^. This transformation generally occurs after the PA has been present for many years, usually decades. The prognosis of salivary malignancies evolving from PA tends to be worse than that of de novo salivary gland malignancies, with a reported 5-years overall survival-rate, as little as 25%^5^. Therefore, the diagnosis of the carcinomatous portion of CXPA is critical in determining the appropriate treatment modality and timing. However, accurate diagnosis of CXPA via biopsy is not easy because it is often difficult to accurately target the carcinomatous portion (CA) of these tumors and this diagnosis is usually possible only when the specimen contains clear adenoma and carcinomatous portions, which tends to be towards the later stages of progression.

Recent molecular analysis of CXPA has shed new light on the mechanisms of carcinogenesis identifying CXPA specific mutations in TP53, PLAG1, and HMGA2^5^. However, for the most part, the landscape of mutational and copy number alterations in CXPA seems to be similar to non-malignant PAs, leaving the molecular processes involved in the transition from PA to CXPA largely unknown^6^.

MicroRNAs (miRNAs) are short non-coding RNAs, involved in the post-transcriptional regulation of gene expression^7^. Dysregulation of miRNA expression has been extensively reported in various types of tumors, including lung, breast, colon, stomach, and thyroid cancers^8^, where it is believed that they play a role in the oncogenic progression or suppression of these cancers. Several studies of salivary gland tumors (SGT) have tried, with varying degrees of success, to identify miRNA signatures for the differential diagnosis of benign and malignant SGTs in an effort to improve preoperative diagnosis using tissue or saliva samples^9^.

**Figure 1:**
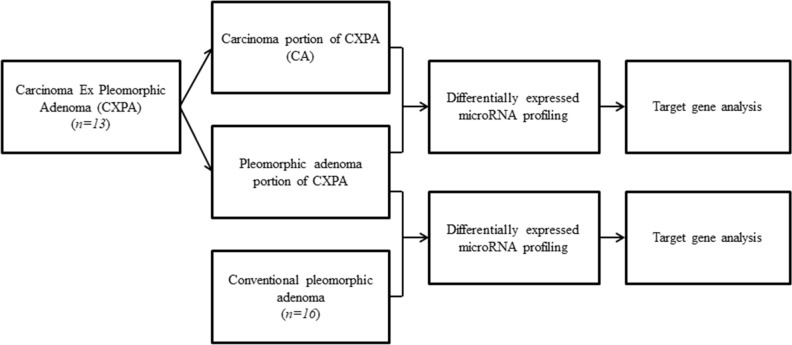
Flowchart describing the selection of the samples and their evaluation in our study.

Here we hypothesized that the presence of the miRNAs involved in malignant transformation of CXPA might help predict the risk of carcinogenesis regardless of histology. Thus, we investigated the candidate miRNAs by comparing the PA and CA samples from CXPA biopsies in an effort to elucidate the pathophysiology of malignant transformation in these cancers. We then compared the miRNA profiles of the PA of CXPA and conventional PA and identified several candidate miRNAs which might be helpful for differential diagnosis between the two (Fig. 1).

## Results

### Clinicopathological data

The mean age of the participants in CXPA group was 53.0 years (ranged 26–71 years) while it was only 44.7 years (ranged 28–75 years) in the conventional PA group. The mean tumor size was 2.6 cm (range 1.4–4.0 cm) in the conventional PA, and 3.5 cm (range 1.2–6.3 cm) in the CXPA groups. Our cases included four SDC, two epithelial-myoepithelial carcinoma, two MEC, three adenocarcinoma NOS, one clear cell carcinoma, and one oncocytic carcinoma presented with a clear CA portion. In addition, risk stratification revealed five patients with high-aggression, eight with low-aggression carcinomas. When we evaluated the extent of the invasion in each sample we were able to identify 5 patients with wide invasive, three with minimally invasive, and five with intracapsular tumors. All of these details are summarized in Table 1.

**Table 1:** Clinicopathologic characteristics.

	Pleomorphic adenoma	Carcinoma ex pleomorphic adenoma	*p* value
Mean age (range)	44.7 (26–71)	53.0 (28–75)	0.120
Sex (male:female)	7:9	6:7	0.897
Size (cm, range)	2.6 (1.4–4.0)	3.5 (1.2–6.3)	0.093
**Subtype of carcinomatous portion**	NA		
Salivary duct carcinoma		4 (30.7%)	
Epithelial-myoepithelial carcinoma		2 (15.4%)	
Mucoepidermoid carcinoma		2 (15.4%)	
Adenocarcinoma		3 (23.1%)	
Clear cell carcinoma		1 (7.7%)	
Oncocytic carcinoma		1 (7.7%)	
**Grade of carcinomatous portion**	NA		
Low aggression		8 (61.5%)	
High aggression		5 (38.5%)	
**Invasiveness**	NA		
Intracapsular		5 (38.45%)	
Minimally invasive		3 (23.1%)	
Widely invasive		5 (38.45%)	
Total	16	13	

*NA* not applicable.

### Identification of differently expressed miRNAs

#### Comparison between the PA and CA in CXPA samples

We identified 2588 miRNAs and 13 differentially expressed miRNAs in the CA and PA of CXPA groups when applying a two-fold change as the threshold (adjusted *p-*value < 0.05). We found five up-regulated (miR-455-3p, miR-140-5p miR-483-5p, miR-125b-5p, and miR-125a-5p) and eight down-regulated miRNAs (miR-21-3p, miR-183-5p, miR-182-5p, miR-425-5p, miR-96-5p, miR-200a-3p, miR-181a-3p, and miR-505-3p) in these CA relative to PA of CXPA samples (Table 2).

**Table 2:** Significantly differentially expressed miRNAs between pleomorphic adenoma portion (CPA) and carcinomatous portion (CA) in carcinoma ex pleomorphic adenoma.

miRNAs, CPA vs CA	Fold change	*p*-value	Adjusted *p*-value
**Up-regulated**			
miR-455-3p	3.409	< 0.001	< 0.001
miR-140-5p	2.91	< 0.001	0.002
miR-483-5p	2.504	0.016	0.025
miR-125b-5p	2.173	0.002	0.003
miR-125a-5p	2.05	0.004	0.007
**Down-regulated**			
miR-21-3p	0.124	0.025	0.037
miR-183-5p	0.148	0.015	0.023
miR-182-5p	0.173	0.012	0.018
miR-425-5p	0.225	0.016	0.049
miR-96-5p	0.246	< 0.001	0.001
miR-200a-3p	0.443	0.006	0.006
miR-181a-3p	0.446	0.03	0.045
miR-505-3p	0.46	0.016	0.024

*CPA* pleomorphic adenoma portion of carcinoma ex pleomorphic adenoma, *CA* carcinomatous portion of carcinoma ex pleomorphic adenoma.

#### Comparision between conventional PA and PA/CA of CXPA samples

We identified a total of 17 differentially expressed miRNAs when comparing conventional PA and PA/CA of CXPA samples (fold change > 2, adjusted *p*-value < 0.05). We identified ten up-regulated miRNAs (let-7a-3p, miR-27a-3p, miR-9-5p, miR-135a-5p, miR-135b-5p, miR-455-5p, miR-218-5p, miR-181d-5p, miR-369-3p, and miR-132-5p) and seven down-regulated miRNAs (miR-196a-5p, miR-193a-3p, miR-193b-3p, miR-29c-3p, miR-331-3p, miR-361-3p, and miR-423-5p) in these evaluations (Table 3).

**Table 3:** Significantly differentially expressed miRNAs between pleomorphic adenoma portion (CPA) in carcinoma ex pleomorphic adenoma (CXPA) and pleomorphic adenoma (BPA).

miRNAs, BPA vs CPA	Fold change	*p*-value	Adjustive *p*-value
**Up-regulated**			
let-7a-3p	3.342	< 0.001	< 0.001
miR-27a-3p	3.136	< 0.001	< 0.001
miR-9-5p	2.669	0.002	0.002
miR-135a-5p	2.63	0.019	0.028
miR-135b-5p	2.201	0.001	0.002
miR-455-5p	2.474	0.033	0.045
miR-218-5p	2.123	0.001	0.004
miR-181d-5p	2.201	< 0.001	< 0.001
miR-369-3p	2.101	0.028	0.028
miR-132-5p	2.041	0.003	0.009
**Down-regulated**			
miR-196a-5p	0.218	0.009	0.022
miR-193a-3p	0.464	0.001	0.004
miR-193b-3p	0.459	< 0.001	< 0.001
miR-29c-3p	0.458	< 0.001	0.002
miR-331-3p	0.424	< 0.001	< 0.001
miR-361-3p	0.49	0.003	0.004
miR-423-5p	0.466	< 0.001	< 0.001

### GO functional enrichment and KEGG pathway analyses

Evaluation of these miRNAs in the biological process category identified several target genes with differential expression in CA and PA of CXPA, which pulled in “apoptotic process”, “cell cycle arrest”, “negative regulation of transforming growth factor beta receptor signaling pathway”, “regulation of transcription from RNA polymerase II promoter”, and “positive regulation of transcription from RNA polymerase II promoter” as their top five GO terms (Table 4). Similarly, when comparing the differentially expressed miRNAs from PA of CXPA and conventional PA, the GO terms for the biological process category for the target genes included “peptidyl-serine phosphorylation”, “positive regulation of transcription, DNA-templated”, “protein phosphorylation”, and “negative regulation of transforming growth factor beta receptor signaling pathway” (Table 4).

**Table 4:** Gene Ontology enrichment of miRNAs which differently expressed in two groups.

Biology process term	Number of involved genes	*p*-value
**Pleomorphic adenoma (CPA) versus carcinomatous portion (CA) in carcinoma ex pleomorphic adenoma**		
GO:0006915 ~ apoptotic process	27	1.39E−04
GO:0007050 ~ cell cycle arrest	11	7.19E−04
GO:0030512 ~ negative regulation of transforming growth factor beta receptor signaling pathway	7	0.00207
GO:0006357 ~ regulation of transcription from RNA polymerase II promoter	20	0.00222
GO:0045944 ~ positive regulation of transcription from RNA polymerase II promoter	35	0.00232
GO:0045165 ~ cell fate commitment	6	0.00254
GO:0071456 ~ cellular response to hypoxia	8	0.00381
GO:0009791 ~ post-embryonic development	7	0.00403
GO:0010468 ~ regulation of gene expression	8	0.00477
GO:0006366 ~ transcription from RNA polymerase II promoter	21	0.00524
**Pleomorphic adenoma (BPA) versus carcinoma ex pleomorphic adenoma (CPA/CA)**		
GO:0018105 ~ peptidyl-serine phosphorylation	15	2.98E−07
GO:0045893 ~ positive regulation of transcription, DNA-templated	28	9.20E−06
GO:0006468 ~ protein phosphorylation	26	9.32E−06
GO:0030512 ~ negative regulation of transforming growth factor beta receptor signaling pathway	9	4.85E−05
GO:0016477 ~ cell migration	14	5.91E−05
GO:0008284 ~ positive regulation of cell proliferation	24	1.08E−04
GO:0045944 ~ positive regulation of transcription from RNA polymerase II promoter	39	1.36E−04
GO:0045892 ~ negative regulation of transcription, DNA-templated	24	2.87E−04
GO:0046777 ~ protein autophosphorylation	12	8.95E−04
GO:0018107 ~ peptidyl-threonine phosphorylation	6	0.00104

Table 5 summarizes the most enriched KEGG pathways for each of the differently expressed miRNAs in both the CA vs PA of CXPA, and PA/CA of CXPA vs conventional PA evaluations. Many cancer-related pathways such as “viral carcinogenesis”, “Hippo signaling pathway”, “p53 signaling pathway”, and “proteoglycans in cancer” were amongst the most highly ranked processes in CA vs PA of CXPA. Interestingly, for the comparison between PA of CXPA and conventional PA, there was a similar pattern in the enriched pathways, including “proteoglycans in cancer”, “ECM–receptor interaction”, Hippo signaling pathway”, “AMPK signaling pathway”, “viral carcinogenesis”, and “TGF-beta signaling pathway”.

**Table 5:** KEGG pathway analysis of the differentially expressed miRNAs and targets in two groups.

KEGG pathway	*p*-value	Numbers of involved genes	Numbers of involved miRNAs
**Pleomorphic adenoma (CPA) versus carcinomatous portion (CA) in carcinoma ex pleomorphic adenoma**			
Viral carcinogenesis	2.54E−12	88	7
Adherens junction	8.65E−10	40	7
Cell cycle	3.23E−09	61	7
Hippo signaling pathway	1.80E−08	60	7
Other types of O-glycan biosynthesis	2.61E−08	11	6
p53 signaling pathway	3.05E−08	40	7
Hepatitis B	5.90E−08	61	7
Proteoglycans in cancer	6.20E−08	80	7
Fatty acid biosynthesis	1.07E−07	3	5
Bacterial invasion of epithelial cells	1.22E−07	37	7
Oocyte meiosis	1.47E−07	51	7
Prostate cancer	9.44E−07	45	7
**Pleomorphic adenoma (BPA) versus carcinoma ex pleomorphic adenoma (CPA/CA)**			
Proteoglycans in cancer	5.23E−14	89	6
ECM–receptor interaction	6.12E−11	34	5
Hippo signaling pathway	2.22E−09	69	6
Prion diseases	2.23E−09	12	5
Bacterial invasion of epithelial cells	9.41E−09	44	6
AMPK signaling pathway	5.16E−07	66	6
Chronic myeloid leukemia	3.29E−06	40	6
Viral carcinogenesis	3.32E−06	83	6
TGF-beta signaling pathway	3.51E−06	42	6
Glioma	3.75E−06	34	6
Fatty acid biosynthesis	4.45E−06	3	4
Adherens junction	1.65E−05	38	6
Thyroid hormone signaling pathway	1.65E−05	57	6

### Target gene prediction, Protein–protein interaction (PPI) networks, and miRNA-Hub Gene network analyses

**Figure 2:**
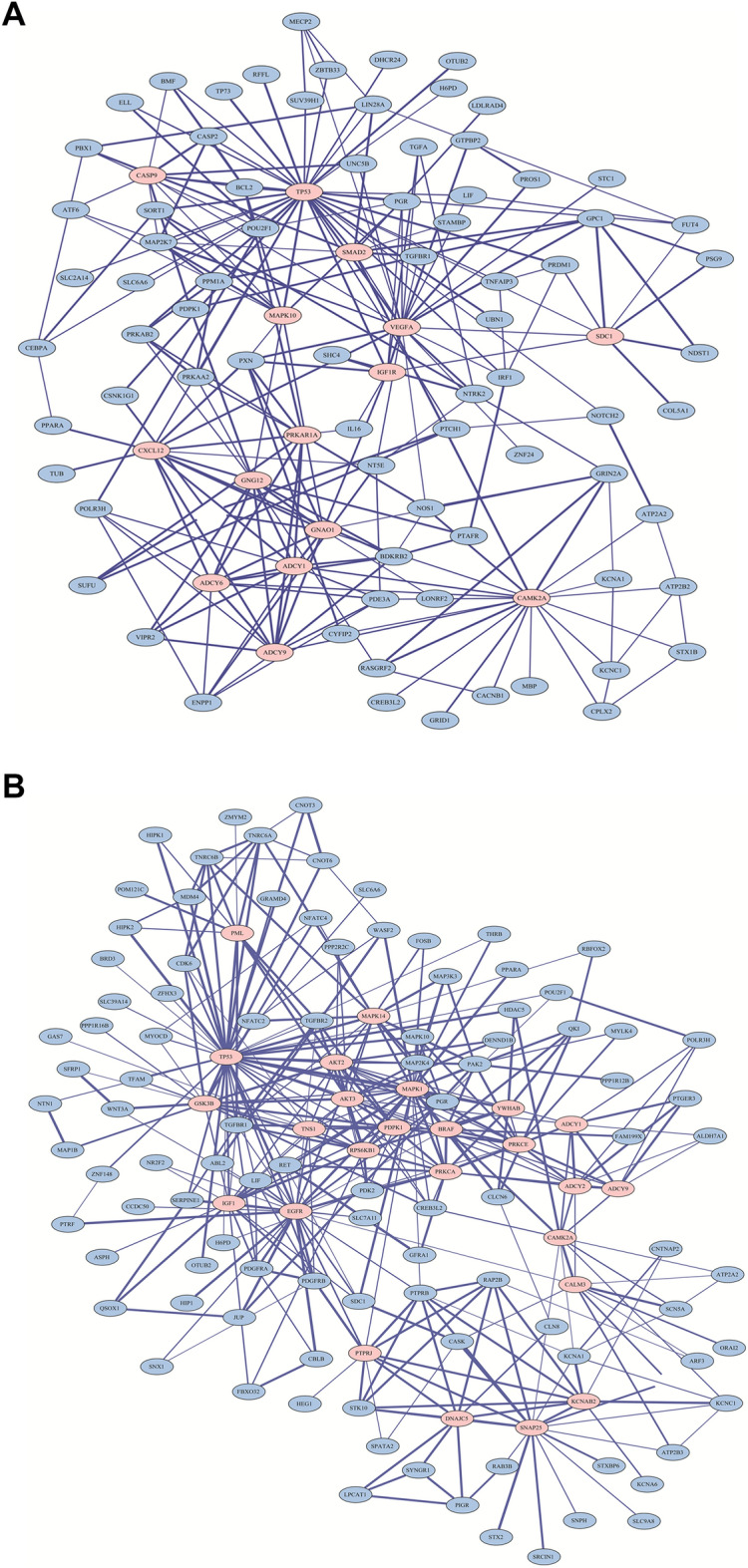
Protein–protein interaction (PPI) network from the STRING database (https://string-db.org/) on Cytoscape software (version 3.8.2, https://cytoscape.org/). (**A**) The interaction network derived from the 15 hub nodes for the targets of the differentially expressed miRNAs between the pleomorphic adenoma portion (PA of CXPA) and carcinoma portion (CA) of carcinoma ex pleomorphic adenoma (CXPA). (**B**) The interaction network derived from 25 hub nodes identified for the targets of the miRNAs with altered expression between conventional pleomorphic adenoma versus PA/CA of CXPA.

A total of 3082 genes were commonly identified by miRWalk, miRanda, RNA22, and Targetscan evaluations, which were then reduced to 383 genes with multiple identifications. We then used the STRING database to identify the PPIs among these 383 targets and identified those proteins that were most likely to interact with more than 10 other proteins and designated these as the hub nodes. Figure 2A shows the PPIs derived from the 15 hub nodes (CASP9, TP53, SMAD2, MAPK10, VEGFA, CXCL12, GNG12, PRKAR1A, IGF1R, SDC1, GNA01, ADCY1, ADCY6, ADCY9, and CAMK2A) for the targets of the differentially expressed miRNAs between PA of CXPA (conventional PA) and CA, with each network finally being constructed using Cytoscape. The most significant revelation from these networks was the identification of TP53 as a central node with more than 33 interactions in these networks.

**Figure 3:**
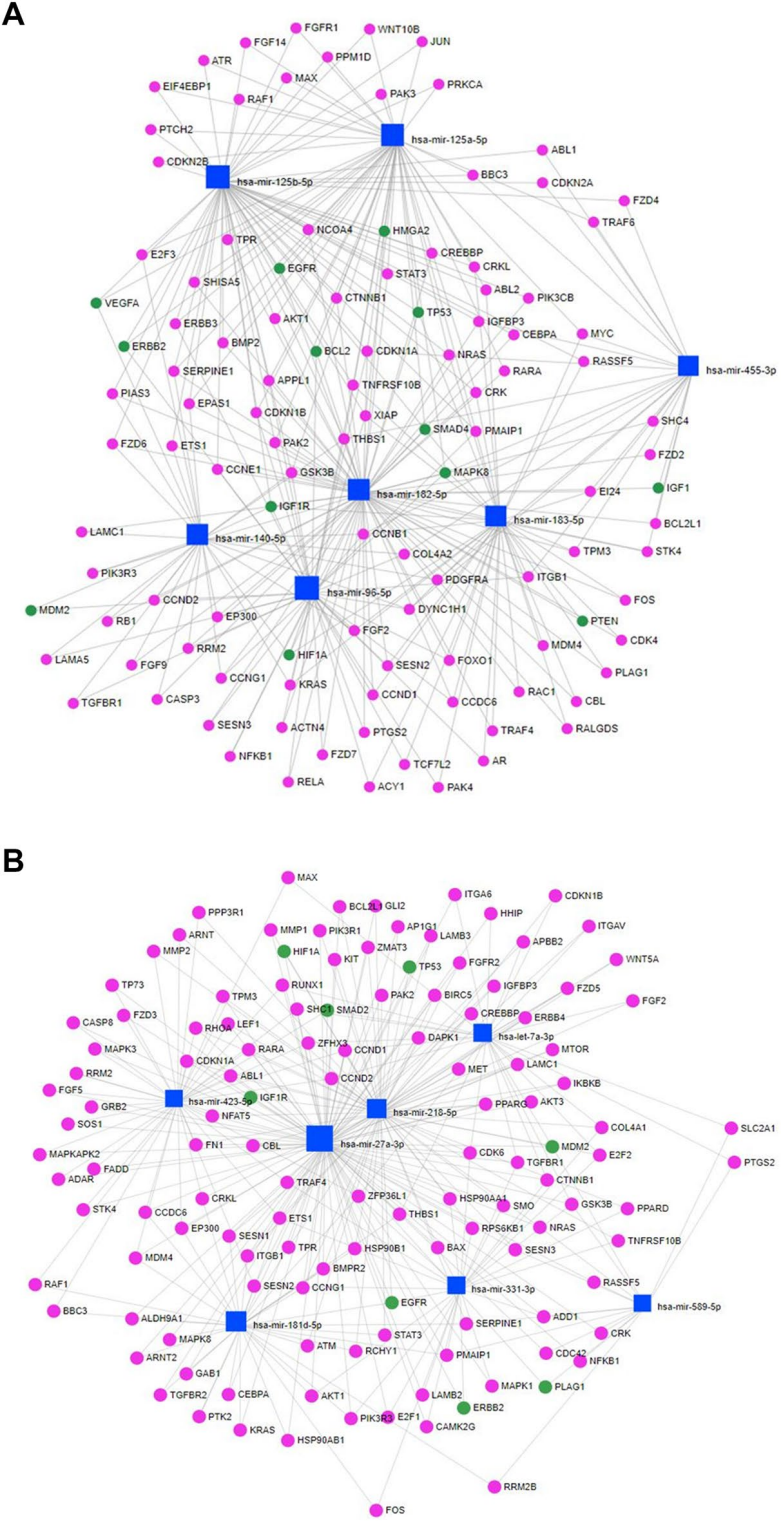
Network of the miRNAs with the target genes related to “pathways in cancer”, “p53 signaling pathway”, and “ErbB signaling pathway” terms from the KEGG database using miRNet software (version 2.0, https://www.mirnet.ca). miRNA-target gene network which derived from the differentially expressed miRNAs from pleomorphic adenoma portion (PA of CXPA) and carcinoma portion of carcinoma ex pleomorphic adenoma (CA) (**A**), and conventional pleomorphic adenoma versus PA/CA of CXPA (**B**).

Figure 2B shows the PPIs for the 25 hub nodes (PML, TP53, MAPK1, MAPK14, AKT2, AKT3, GSK3B, TNS1, PDPK1, BRAF, YWHAB, ADCY1, ADCY2, CAMK2A, CALM3, PRKCA, PRKCE, RPS6KB1, TNS1, IGF1, EGFR, PTPRJ, DNAJC5, KCNAB2, and SNAP25) identified for the targets of the differentially expressed miRNAs between conventional PA and PA of CXPA (CA). Interestingly, TP53 was once again identified as the central node with more interactions than any other target. Based on these findings, we created a final miRNAs-target gene network focused on their connections to the “pathways in cancer”, “p53 signaling pathway”, and “ErbB signaling pathway” terms obtained from the KEGG database (Fig. 3A,B).

## Discussion

CXPA is an uncommon SGT with relatively aggressive features that requires complete excision to prevent progression making its preoperative diagnosis a critical factor in improving clinical outcome. However, its mixed cellularity and the heterogeneity of benign and malignant components can make the detection of its CA component challenging, thus reducing the cytodiagnostic accuracy of CA in CXPA to roughly 50% and increasing false-negative diagnosis of PA to 38.5%^10^. Several studies have tried to identify novel biomarkers to facilitate better diagnostic accuracy in SGT. Many have evaluated the use of miRNAs to distinguish between patients with malignant and benign SGT using both tissue and circulating miRNA targets (saliva or blood). This is because miRNA profiling is expected to create significant diagnostic, prognostic, and/or predictive value, and to help uncover the etiological role of specific miRNAs in cancer initiation, progression, and/or metastasis in various cancers^11–16^. Numerous studies have identified a wide range of differentially expressed miRNAs in both benign and malignant SGTs, and a recent study attempted to create miRNA signatures for the diagnosis of 24 SGT patients (10 benign, and 14 malignancies) using a panel of 798 miRNAs and further suggested that 46 of them are likely to be differently expressed in benign and malignant tumors and may act as potential biomarkers for their differential diagnosis^9^. Another study evaluating 38 malignant and 29 benign parotid gland tumors identified four miRNA combinations (miR-132, miR-15b, miR-140, and miR-223) that could discriminate between malignant and benign parotid tumors using saliva samples^17^. On the other hand, a recent meta-analysis concluded that altered miRNA expression in SGTs may be useful for prognostication though their diagnostic value is still limited owing to the small sample sizes and pathological heterogeneity^18^.

Most of these studies include various kinds of malignant SGTs, while our study tried to identify differentially expressed miRNAs specifically associated with the PA and CA portions of CXPA tumors. Our study also compared the expression of these miRNAs between PA of CXPA and conventional PA in an attempt to understand the pathogenesis of malignant transformation in CXPA, and identify those precancerous miRNA changes that may facilitate the conversion from conventional PA to PA of CXPA.

Interestingly, both miR-21 and miR-96-5p are up-regulated in CA compared to PA of CXPA and both are known to act as onco-miRNAs in head and neck squamous cell carcinoma (HNSCC)^19^. This is further supported by the fact that miR-21 is commonly up-regulated in various malignant SGTs when compared to their benign tumor counterparts^9,20^. In addition, miR-96-5p forms part of the miR-96/182/183 cluster and its overexpression is also known to be found in patients with p53 mutations, where it acts to support cell migration and chemoradiation resistance via its targeting of PTEN and activation of the PI3K-AKT signaling pathway in HNSCC^19^. miR-425-5p is another well-known onco-miRNA which is upregulated in breast cancers where it targets PTEN^21^, and in MEC tissues, where the function remains unidentified^22^.

miR-125a-5p, miR-125b-5p, and miR-140-5p, which are down-regulated in the CA tissues when compared to PA of CXPA samples, are also known for their tumor suppressor activities in HNSCC^19^. Of these, both miR-125b-5p and miR-140-5p are also known to be significantly down-regulated in malignant SGT samples relative to their benign counterparts^9^. mir-455-3p has also been reported to act as a tumor suppressor gene downstream of TP53 inducing pro-apoptotic activity in pancreatic^23^, osteosarcoma^24^, and breast cancer^25^.

Unlike previous studies, we identified seven miRNAs (miR-196a-5p, miR-193a-3p, miR-193b-3p, miR-29c-3p, miR-331-3p, miR-361-3p, and miR-423-5p), which demonstrated specific differential expression in CA and PA of CXPA tissues when compared to conventional PA samples. Interestingly, some differentially expressed mRNAs (miR-196a-5p, miR-193b-3p, miR-423-5p, and miR-361-3p) were reported to be associated with oral cavity squamous cell carcinoma^26–28^. Moreover, miR-193a-3p was reported to be associated with MEC^22^, and miR-331-3p has been linked to AdCC^29^. These findings are particularly significant because, although PA of CXPA and conventional PA present with nearly indistinguishable histological profiles, their onco-miRNA profiles are significantly different. These unique results suggest that these miRNAs may be associated with some of the pre-malignant changes in these samples even when the pathology remains benign. Thus, we suggest that these pre-malignant miRNAs may facilitate better diagnostic accuracy for fine needle aspiration cytology or core needle biopsy in clinical settings. Based on these findings, we can infer that although the PA of CXPA was biopsied based on its heterogeneity of benign and malignant portions, the specific miRNAs can be expected to predict the possibility of malignancy.

Furthermore, the down-regulated miRNAs in PA/CA of CXPA relatively to the conventional PA samples were also shown to be well-known tumor suppressor miRNAs, including the let-7 family transcripts, miR-9-5p, miR-135a-5p and miR-135b-5p, amongst others. miR-9-5p, miR-135a-5p, and miR-135b-5p, were all shown to be down-regulated in malignant vs. benign SGT samples^9^, and the differential expression of both miR-132-5p and miR-140 was consistent with the results of other studies^17^.

GO enrichment and KEGG pathway analyses revealed that these differentially expressed transcripts were all linked to the regulation of several critical oncogenic signaling pathways, such as the p53 signaling pathway. Moreover, GO enrichment and KEGG pathway evaluations of the differentially expressed miRNAs from PA of CXPA vs. conventional PA also revealed their involvement in signal regulation with these transcripts being linked to various critical signaling pathways including the AMPK and TGF-beta signaling pathways. PLAG1 or HMGA2 fusions are also useful biomarkers for distinguishing between PA and various other pathologies suggesting that CXPA tissues are also likely to encode these PA-specific gene fusions^30^. In addition, the amplification of MDM2, mutations in TP53, gains and amplifications of MYC, Epidermal growth factor receptor (EGFR), HGF-A (scatter factor), c-Met (a proto-oncogene), Transforming growth factor alpha (TGF α), Fibroblast growth, factors (FGF)-2, and ErbB2 (HER2), amongst others have all been hypothesized to play a role in the progression and invasion of CXPA^31–34^. Thus, it was not surprising that our PPI evaluations included several of these genes, including ErbB2, TP53, HMGA2, PLAG1, HIF1A, EGFR, VEGFA, and MDM2, into the miRNA-Hub gene networks created for both conditions. The most significant hub gene in both the networks was found to be TP53, known to be implicated in the malignant transformation of CXPA. In addition, another recent study reported the molecular events underlying PA’s malignant transformation in one patient who experienced three bouts of recurrent disease before finally receiving a CXPA diagnosis, where they identified the sequential mutation of TP53 in these recurrent tumors, all of which are consistent with our results^35^. It must be noted that due to the rarity of this disease, we were forced to adopt a retrospective study design and were limited to a small cohort. This limited our ability to perform any validations in a second independent cohort. However, to the best of our knowledge, there have been no other studies evaluating the independent profiles of the PA and CA portions of CXPA nor any comparing the profiles of PA of CXPA and conventional PA. Given this we believe that our results offer a significant insight into the roles of miRNA during CXPA carcinogenesis. Further studies would be required for the validation of these target miRNAs in CXPA and to reveal their possible relationship with other well-known genes involved in CXPA such as PLAG-1 and HMGA2.

In conclusion, we identified several differentially expressed miRNAs in both the CA and PA of CXPA. Specifically, our analysis identified the well-known onco-miRNAs, miR-21 and miR-96-5p, and tumor suprressors miR-125a-5p, miR-125b-5p, and miR-140-5p to have altered expression in the malignant component of CXPA (CA) compared to the benign component (PA of CXPA). In addition, we found that they are related to TP53 and cancer-related pathways using target gene prediction and gene pathway analyses. Furthermore, our evaluations identified a handful of unique miRNAs that may help to differentiate between conventional PA and PA of CXPA, which may be valuable in the development of novel liquid biopsy tools. Taken together these results suggest that miRNAs affect tumor suppressor genes such as p53 during the malignant transformation of CXPA, and that there is a possibility that PA of CXPA may have the potential to progress to CXPA via the differential regulation of several key miRNAs.

## Materials and methods

### Patients and materials

This study was carried out in accordance with the tenets of the Declaration of Helsinki for research regarding human subjects. This present study was approved by the Seoul National University Institutional Review Board for application at both Bundang Seoul National University Hospital (No.B-1905-540-304) and Dongtan Sacred Heart Hospitals, Hallym University (No.2019-04-298). Bundang Seoul National University Hospital committee waived the need for written informed consent on 11 samples obtained before 2013, and another 18 subjects gave their written informed consent. All methods were carried out in accordance with relevant guidelines and regulations.

**Figure 4:**
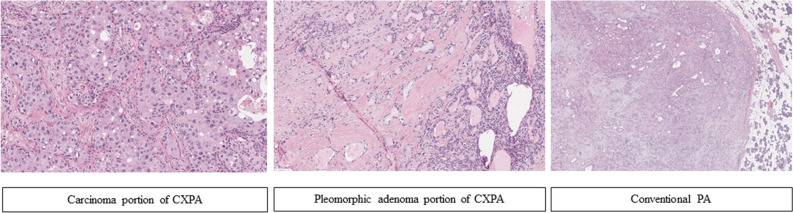
Histopathologic findings of the carcinomatous portion (CA), pleomorphic adenoma portion (PA) of carcinoma ex pleomorphic adenoma (CXPA) (H & E, 100x) and conventional PA (H & E, 20x).

The sample collection was done as described in Fig. 1. A total of 13 CXPA and 16 conventional PA patients who underwent surgical resection at one of these two hospitals between 2010 and 2019 were enrolled in this study based on their pathological presentation. Their original hematoxylin–eosin (H and E)–stained slides were reviewed by the specialist head and neck pathologist (H.K), and the diagnoses in each case were categorized following the World Health Organization (WHO)’s Classification of Salivary Gland Tumors^36^. All CXPA were classified according to their invasiveness (intracapsular, minimally, and widely invasive) and histological subtype. As per the risk stratification of SGTs based on the WHO (2017) classification our cases include low aggression tumors namely epithelial-myoepithelial carcinoma, clear cell carcinoma, oncocytic carcinoma, adenocarcinoma NOS, and low grade MEC; the high aggression tumor, SDC, and high grade MEC. Macro-dissection of PA and CA portion of CXPA was performed under microscopic marking. Figure 4 shows all the pathologic findings of CA, PA portion of CXPA, and conventional PA.

### RNA isolation

Total RNA was extracted from paraffin-embedded tissues using Trizol reagent (Invitrogen, Carlsbad, CA, USA) according to the manufacturer’ instructions. CXPA tissues were deparaffinization with xylene and the sections stained with H & E before the PA and CA portions of CXPA were selected and carefully dissected to minimize cross contamination. RNA quality was assessed using an Agilent 2100 bioanalyzer and an RNA 6000 Pico Chip (Agilent Technologies, Amstelveen, The Netherlands), and RNA quantification was performed using a NanoDrop 2000 Spectrophotometer system (Thermo Fisher Scientific, Waltham, MA, USA).

### Library preparation and sequencing

Sequencing libraries were generated for both the control and test RNAs using the NEBNext Multiplex Small RNA Library Prep kit (New England BioLabs, Inc., USA) according to the manufacturer’ instructions. Briefly, 1 µg of total RNA from each sample was ligated to the adaptors before being transcribed to cDNA using reverse-transcriptase and adaptor-specific primers. Libraries were then amplified by PCR and prepared for sequencing using a QIAquick PCR Purification Kit (Qiagen, Inc, German) and AMPure XP beads (Beckman Coulter, Inc., USA). The yield and size distribution of the small RNA libraries were assessed using the Agilent 2100 Bioanalyzer (Agilent Technologies, Inc., USA) and high-throughput sequences were generated by single-end 75 sequencing using a NextSeq500 system (Illumina, San Diego, CA., USA).

### Data analysis

Sequence reads were mapped using bowtie2 software (v2.4.2, http://bowtie-bio.sourceforge.net/bowtie2/) and the final bam file for evaluation (alignment file) was created. Mature miRNA sequences were then used as a reference for mapping. The read counts for each mature miRNA sequence were extracted from the alignment file using bedtools (v2.25.0) and Bioconductor programmed to use R (version 3.2.2; R development Core Team, 2011) as its base. Read counts were then used to determine the expression level of each miRNA and the quantile normalization method for between sample comparisons. We then identified the miRNA targets using the miRWalk 2.0 database (http://zmf.umm.uni-heiderlberg.de/apps/zmf/mirwalk2/)^37,38^. Gene ontology (GO) and Kyoto Encyclopedia of Genes and Genomes (KEGG) pathway enrichment analyses were then applied using the Database for Annotation, Visualization, and Integrated Discovery (DAVID) online tool (http://david.abcc.ncifcrf.gov/)^39,40^ and specific GO and KEGG pathway terms were identified using a *p-*value threshold of < 0.01. The protein–protein interaction data were obtained from the STRING database (http://string-db.org/) and visualized using Cytoscape software (version 3.8.2, https://cytoscape.org/). These analyses were then combined to create the miRNA-target gene networks for these samples using miRNet software (version 2.0, https://www.mirnet.ca).

### Statistical analysis

All statistical analyses were completed in R (version 3.5.1) using R studio (version 1.1.383, https://www.rstudio.com). T-test was used to evaluate the differential expression between different sample types and a *p-*value of < 0.05 was considered statistically significant. The R “stats” package was used for all Benjamini–Hochberg corrections.

### Ethics approval

The study was approved by the Committee on Seoul National University Bundang Hospital (No. B-1905-540-304) and Dongtan Sacred Heart Hospital (No. 2019-04-298), Republic of Korea.

## Data Availability

Data has been deposited to the Gene Expression Omnibus under the accession of GSE205670 (www.ncbi.nlm.nih.gov/geo).
